# Mobilization of *bla*_VIM_ genes via the Tn6292 transposon among carbapenem-resistant *Enterobacter cloacae* complex isolates from colonized patients in a Spanish hospital

**DOI:** 10.1128/spectrum.04162-25

**Published:** 2026-07-21

**Authors:** Esther Ríos, Iciar Rodríguez-Avial, Rodrigo López, Mercedes Martínez, Fernando González, Alberto Delgado-Iribarren, Esther Culebras

**Affiliations:** 1 Servicio de Microbiología Clínica, Instituto Medicina Laboratorio (IML), Fundación para la Investigación Biomédica del Hospital Clínico San Carlos (IdISSC), Hospital Clínico San Carlos, Madrid, Spain; 2 Departamento de Medicina (Microbiología), Facultad de Medicina, Universidad Complutense, Madrid, Spain; 3 Unidad De Apoyo Metodológico a la Investigación (UAMI), Fundación para la Investigación Biomédica del Hospital Clínico San Carlos (IdISSC), Hospital Clínico San Carlos, Madrid, Spain; National Institute of Science Education and Research, Jatni, Odisha, India

**Keywords:** *Enterobacter cloacae* complex, carbapenemase genes, colonizers, whole-genome- sequencing, *bla*_VIM_, ST93, ST78, Tn6292

## Abstract

The aim of this study was to perform molecular characterization of the carbapenem-resistant *Enterobacter cloacae* complex (ECC) isolates from colonized patients in a hospital using whole-genome sequencing (WGS) technology. As part of routine surveillance for multidrug-resistant bacterial colonization, 21 ECC isolates were recovered from patients at San Carlos Hospital in Madrid (Spain) between December 2020 and November 2024. WGS was used to determine their genetic relatedness. Furthermore, species identification, sequence type (ST), resistome, plasmid content, and flanking mobile genetic elements (MGEs) of the carbapenemase genes were derived from the WGS data. The most prevalent carbapenemase gene identified was *bla*_VIM-1_ (*n* = 18, 85.7%), with other notable genes including *bla*_KPC-2_ (*n* = 1, 4.8%), *bla*_KPC-3_ (*n* = 1, 4.8%), and *bla*_OXA-48_ (*n* = 1, 4.8%). Several *bla*_ACT_ and *bla*_ESBL_ variants were also found among the carbapenem-resistant ECC isolates. All of them carried at least one *bla*_ACT_ gene, with *bla*_ACT-7_ (11/21) and *bla*_TEM_-type (14/21) genes being the most common AmpC and ESBL-encoding genes, respectively. Additionally, two isolates exhibited the presence of the *mcr-9* gene. Overall, *E. hormaechei* subsp. *steigerwaltii* (ST93), followed by *E. hormaechei* subsp. *hoffmanii* (ST78 and ST50), were the predominant species and STs circulating among the carbapenem-resistant ECC strains. The *bla*_VIM-1_ gene was part of class 1 integrons located within a Tn3-family transposon, Tn6292. *bla*_KPC_ and *bla*_OXA-48_ were linked to Tn4401 and Tn1999 transposons, respectively. In conclusion, the presence of the *bla*_VIM_ within a transposon Tn6292 enhances its mobility across bacterial genomes, underscoring the value of high-throughput sequencing in monitoring the spread of carbapenem-resistant ECC isolates.

**IMPORTANCE:**

This study highlights why monitoring the spread of antibiotic-resistant bacteria in hospitals is critical. By analyzing the complete DNA of carbapenem-resistant bacteria, antibiotics were considered a last line of treatment. We found that the resistance genes are not isolated. Instead, they are embedded within mobile elements called transposons. This means that they can “jump” between different bacteria, accelerating the spread of resistance. These findings emphasize the importance of high-resolution genomic technologies to track and control the spread of these dangerous bacteria in clinical settings, helping preserve the effectiveness of life-saving treatments.

## INTRODUCTION

Species of the *Enterobacter cloacae complex* (ECC) are a group of facultative anaerobic, non-spore-forming Gram-negative bacteria belonging to the family *Enterobacterales*. These microorganisms are part of the healthy gut microbiota of humans and animals. The ECC exhibits remarkable diversity, encompassing numerous species and subspecies. Currently, 10 species and 22 clades have been classified within the ECC (1). They are also members of the ESKAPE group, six major bacterial pathogens—*Enterococcus faecium*, *Staphylococcus aureus*, *Klebsiella pneumoniae*, *Acinetobacter baumannii*, *Pseudomonas aeruginosa*, and *Enterobacter* species—that are leading causes of hospital-acquired (nosocomial) infections. These bacteria also display multidrug resistance and virulence.

The intrinsic resistance of ECC to multiple β-lactam antimicrobials is largely explained by the constitutive expression of the *ampC* gene located on the chromosome (2, 3). These microorganisms can overproduce AmpC β-lactamases either through de-repression of the chromosomal gene or via acquisition of a transferable *ampC* gene carried on plasmids or other mobile genetic elements, thereby conferring antibiotic resistance (4).

In addition to intrinsic β-lactam resistance, multiple molecular mechanisms may coexist, including the production of other β-lactamases (such as extended-spectrum β-lactamases [ESBLs] and carbapenemases), as well as impaired outer membrane permeability (2, 3). Different types of ESBLs and carbapenemases have been identified in drug-resistant strains of ECC. The majority of ESBLs belong to class A (Ambler classification) with predominant enzymes including TEM, SHV, and CTX-M (5). The main carbapenemases found in carbapenem-resistant *Enterobacteriaceae* are Ambler class A (KPC), class B metallo-β-lactamases (IMP, VIM, and NDM), and class D oxacillinases (OXA-48-like) enzymes (6).

Furthermore, ECC has become a reason of concern as a cause of hospital-acquired infections, especially multi-resistant strains. Hospital settings are vulnerable to outbreaks caused by patient-to-patient or environmental transmission (4, 7). Numerous cases and outbreaks of carbapenemase-producing ECC isolates have been widely reported across the globe (8–13), with VIM, NDM, and OXA-48 representing the most prevalent types in Europe (2, 5). Current literature indicates that the emergence and spread of these pathogens is due to a combination of high genetic diversity and acquisition of resistance genes via plasmids and transposons (3, 14). Genome analysis and molecular epidemiology are fundamental tools for confirming suspicion of an outbreak at an early stage, as well as for surveillance and infection control. Hence, the aim of the present study was to characterize the carbapenemase-producing ECC isolates from colonized patients using whole-genome sequencing (WGS) technology.

## MATERIALS AND METHODS

### Bacterial isolates

Between December 2020 and November 2024, a total of 21 carbapenemase-producing ECC isolates were collected from patients admitted to San Carlos Hospital (Madrid, Spain). These strains were recovered through routine surveillance for multi-resistant bacterial colonization. Following the recommendations of the Spanish National Epidemiological Surveillance Network (RENAVE) (Spain) (15), screening for colonization by the carbapenemase-producing ECC was performed using both passive and active strategies.

Passive screening was performed in any hospitalized patient with a current infection caused by ECC or with a documented history of infection or colonization within the preceding 12 months. In these cases, pharyngeal, perineal, and rectal swab samples were processed weekly until two consecutive screenings were negative.

Active screening was limited to patients of any age admitted to high-risk units such as the intensive care unit (ICU). During outbreaks or periods of high endemicity, active screening was extended to the affected wards. In these cases, rectal swabs were obtained at admission and weekly throughout hospitalization. Patients with positive cultures were subsequently managed according to the passive screening protocol.

Samples obtained from both passive and active screening were cultured on MacConkey agar (bioMérieux, France) with imipenem, cefotaxime, ciprofloxacin, and gentamicin disks (BBL Sensi-Disc, BD, Maryland, USA), as well as on selective chromogenic media, including ChromID ESBL and ChromID CARBA SMART biplate (bioMérieux, France).

Bacterial identification was performed by MALDI-TOF mass spectrometry (Bruker Daltonics) and carbapenemase detection was confirmed using both the Carba NP test and NG Carba5 (NG Biotech, Guipry, France).

### DNA extraction and whole-genome sequencing (WGS)

Genomic DNA of isolates was extracted using a microbiome DNA extraction kit (Altair Health, Spain) in accordance with the manufacturer’s instructions. DNA quantity was measured using a Qubit 2.0 fluorometer (Thermo Fisher Scientific, Waltham, MA, USA). Rapid Barcoding Kit 24 V14 (SQK-RBK114.24) (Oxford Nanopore Technologies, UK) was used for library preparation. WGS was performed on a GridION using R10.4.1 flow cells and nanopore technology on the MinION platform (Oxford Nanopore Technologies, UK).

### Bioinformatic analysis

The initial assembly and bioinformatics analysis of the genomic sequences were carried out using Epidemiological Surveillance NGS (Altair Health Biotechnology, Madrid, Spain). This software includes several tools for filtering (Filtlong), correction and assembly (Fly and Medaka), and gene comparison (TORMES version 1.3.0). TORMES is an automated pipeline that relies on many different software and databases to generate comprehensive genomic characterization reports. It provided taxonomic identification (Kraken2), genomic annotation (Prokka), multilocus sequence typing (MLST), and antimicrobial resistance genes (ResFinder) (16).

The close genetic environment of the carbapenemase genes was assessed using online tools and databases available at the Center for Genomic Epidemiology (CGE) website (17), such as MobileElementFinder v1.0.3 for the investigation of mobile genetic elements and PlasmidFinder 2.1 for plasmid typing. In addition, the DNA sequences were further analyzed by using BacAnt (18). Integrons were classified according to INTEGRALL (19). A graphic map was generated by Clinker (v0.0.31) to illustrate the predominant mobile genetic structures responsible for the dissemination of carbapenemase-encoding genes.

Additionally, the following databases and typing schemes were also employed: BLAST (20), the National Center for Biotechnology Information (NCBI), and the *Enterobacter cloacae* multilocus sequence typing (MLST) databases (21).

Maximum-likelihood phylogeny was carried out with Snippy (v4.6.0) with *Enterobacter hormaechei* (NC_021046.1) as the reference strain and removing recombination events with Gubbins (v2.4.1). Finally, the single-nucleotide polymorphism (SNP)-based phylogenetic tree was constructed after 1,000 bootstrap replicates with the IQ-Tree (v2.0.7).

## RESULTS

### Identification and characteristics of bacterial isolates

Of the 21 carbapenem-resistant ECC strains collected, the majority (*n* = 12, 57.14%) were isolated from rectal and perineal samples, followed by urinary (*n* = 5, 23.8%) and respiratory specimens (*n* = 3, 14.3%). The remaining one isolate was obtained from a submandibular abscess.

The 21 ECC isolates were identified as *E. hormaechei* (*n* = 19; 90.5%) and *E. kobei* (*n* = 2; 9.5%). There were four subspecies of *E. hormaechei* with *Enterobacter hormaechei* subsp. *steigerwaltii* (*n* = 10) being the most predominant, followed by *E. hormaechei* subsp. *hoffmannii* (*n* = 5), *E. hormaechei* subsp. *hormaechei* (*n* = 3), and *E. hormaechei* subsp. *xangfangensis* (*n* = 1).

### Resistance genes

**Fig 1:**
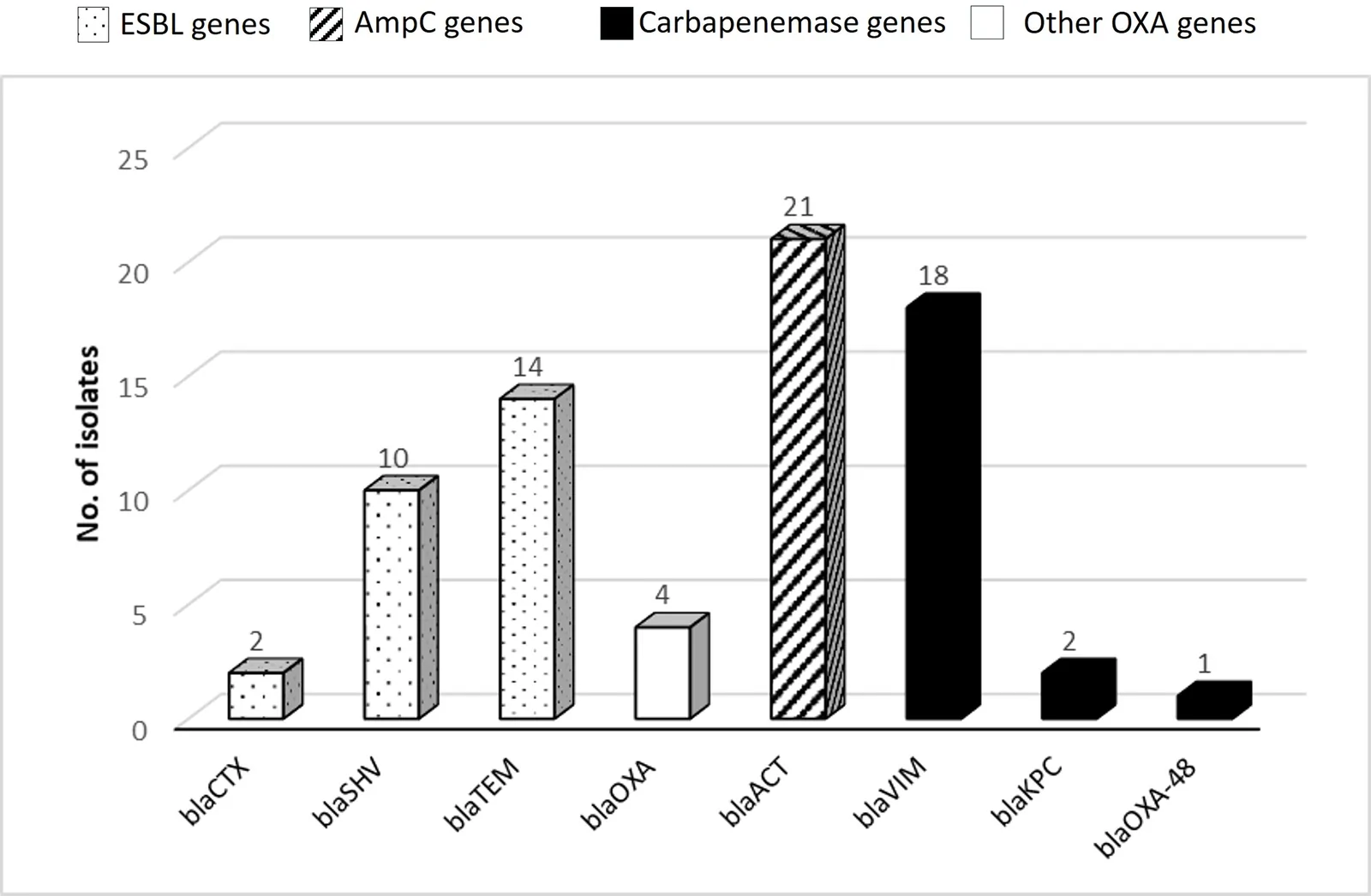
Distribution of the *bla* genes identified among the 21 *Enterobacter cloacae* complex isolates.

WGS revealed several types of genes *bla* encoding beta-lactamases among the 21 carbapenem-resistant ECC isolates. The distribution of genes according to the enzyme they encode is shown in Fig. 1. All strains harbored one carbapenemase gene, being the most prevalent *bla*_VIM_ (*n* = 18, 85.7%), followed by *bla*_KPC_ (*n* = 2, 9.5%) and *bla*_OXA-48_ (*n* = 1, 4.8%). *bla*_VIM-1_ was the only VIM variant detected, whereas the *bla*_KPC-2_ and *bla*_KPC-3_ variants were identified in two isolates, respectively.

Additional OXA-type β-lactamase genes in the genome of four isolates included OXA-1 (*n* = 1), OXA-9 (*n* = 1), and OXA-10 (*n* = 2).

**Fig 2:**
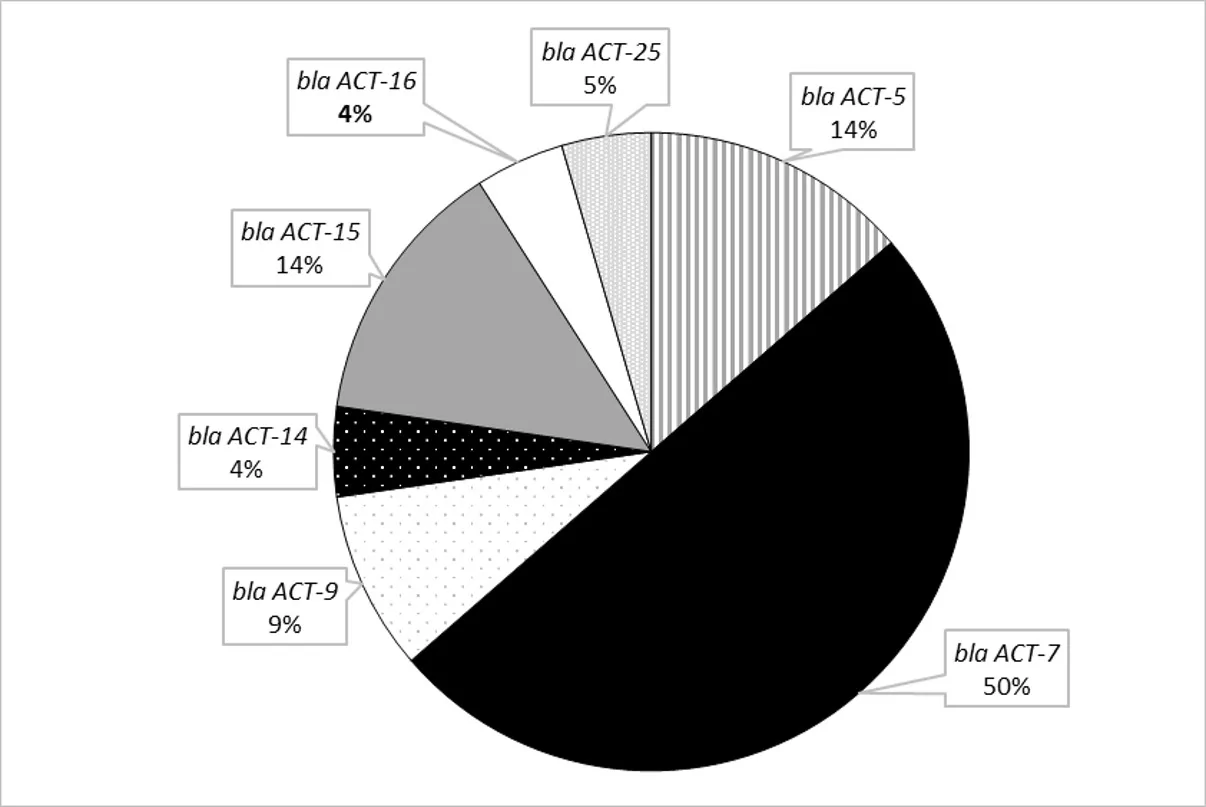
The different types of *bla*AmpCs genes among carbapenem-resistant *Enterobacter cloacae* complex.

ESBL and AmpC encoding genes were also observed among the 21 carbapenem-resistant ECC isolates. A total of 17 (81%) carried *bla*_ESBL_ genes, with *bla*_TEM-1_ being the most common (*n* = 14, 66.7%). Other ESBL genes included *bla*_SHV-12_ (*n* = 10) and *bla*_CTX-M-15_ (*n* = 2). Regarding AmpC genes, all strains carried at least one *bla*_ACT_ gene, with *bla*_ACT-7_ being the most frequent, identified in 11 out of 21 isolates (50%) (Fig. 2). Notably, one isolate co-harbored two *bla*_ACT_ variants (*bla*_ACT-5_ and *bla*_ACT-25_).

### Multilocus sequence typing

MLST analysis identified 10 distinct STs for the 21 carbapenemase-carrying ECC isolates. Table 1 summarizes the sequence types (STs) and antimicrobial resistance genes associated with each clone. ST93 was the predominant ST type, accounting for 43% (*n* = 9), followed by ST78 (*n* = 3, 14.3%) and ST50 (*n* = 2, 9.5%). Notably, three strains (harboring *bla*_VIM_) were classified as ST190, ST758, and ST145, double-locus variants (DLVs) of the prevalent clones ST93 and ST78.

**TABLE 1:** Characteristics of clones identified in *Enterobacter cloacae* complex strains according to MLST^*a*^

Sequence type (ST) (*N*)	Species identification (*N*)	Resistance genes (No. of isolates)			Location of carbapenemase gene	
		Carbapenems	β-lactams	Others	Plasmid type (*N*)	Mobile genetic elements (*N*)
ST93 (9)	*E. hormaechei* subsp. *steigerwaltii* (8)	*bla-_VIM-1_* (8)	*bla_ACT-7_* (8) *bla_TEM-1B_* (8) *bla_SHV-12_* (7)	*fosA-7* (8); *qnrS-1 (6)*; *aac(3)-IId* (8); *sul1* (8); *sul2* (5); *dfrB1* (8); *dfrA1* (5); *dfrA12* (8)	ND (8)	Tn6292 (8), In3103 (6), In654 (2)
	*E. hormaechei* subsp. *hormaechei* (1)	*bla-_KPC-2_*	*bla_ACT-7_* *bla_TEM-1B_*	*fosA-7*; *qnrS-1*; *aac(3)-IId*; *sul1*; *dfrA12*	IncP	Iskpn27
ST190 (DLV ST93) (1)	*E. hormaechei* subsp. *steigerwaltii*	*bla-_VIM-1_*	*bla_ACT-7_* *bla_TEM-1B_* *bla_OXA-10_*	*fosA-7*; *qnrA-1*; *ant(2'')-Ia*; *aph(3')-Ia*; *aac(6')-Ib3*; *sul1*; *dfrB1*; *dfrA14*	ND	Tn6292, In1114
ST78 (3)	*E. hormaechei* subsp. *hormaechei* (1)	*bla-_VIM-1_* (3)	*bla_ACT-5_* (1) *bla_SHV-12_* (1)	*fosA-1* (1); *qnrS-1* (1); *dfrA14* (1); *dfrA15* (1); *sul1* (1); *dfrB1* (1)	IncL/M (1), ND (2)	Tn6292 (3), In3103 (1), In654 (1), In476 (1)
	*E. hormaechei* subsp. *hoffmannii* (2)		*bla_ACT-5_* (2) *bla_SHV-12_* (2) *bla_TEM-1B_* (1) *bla_CTX-M-15_* (1)	*fosA-1* (2); *qnrS-1* (2); *dfrA14* (2); *dfrA15* (1); *sul1* (2); *dfrB1* (1)		
ST145 (DLV ST78) (1)	*E. hormaechei* subsp. *hoffmannii*	*bla-_VIM-1_*	*bla_ACT-14_* *bla_SHV-12_*	*mcr-9*; *fosA-7*; *qnrS-1*; *sul1*; *dfrB1*	IncL/M	Tn6292, In624
ST50 (2)	*E. hormaechei* subsp. *hormaechei*	*bla-_VIM-1_* (2)	*bla_ACT-15_* (2)	*fosA-7* (2); *qnrS-1* (2);* sul1* (2);* dfrA14* (2)	IncN (2)	Tn6292 (2), In1114 (2)
ST90 (1)	*E. hormaechei* subsp. *hormaechei*	*bla-_VIM-1_*	*bla_ACT-15_* *bla_TEM-1B_* *bla_OXA-10_*	*fosA-7*; *qnrA-1*; *aac (3)-IId*; *sul1*; *dfrA14*; *dfrB1*	ND	Tn6292, In1114
ST758 (DLV ST93) (1)	*E. hormaechei* subsp. *steigerwaltii*	*bla-_VIM-1_*	*bla_ACT-7_*	*fosA-7*; *sul5*	ND	In1114
ST114 (1)	*E. hormaechei* subsp. *xiangfangensis*	*bla-_VIM-1_*	*bla_ACT-16_* *bla_ACT-25_* *bla_TEM-1B_ bla_CTX-M-15_* *bla_OXA-1_*	*fosA-7*; *qnrB-1*; *aac (3)-IIa*; *aac(6')-Ib-cr*; *aac(6')-Ib3*; *sul1*; *sul2*; *dfrA1*; *dfrA14*	ND	Tn6292, In654
ST54 (1)	*E. kobei*	*bla_KPC-3_*	*bla_ACT-9_* *bla_TEM-1A_ bla_OXA-9_*	*mcr-9*; *fosA-1*	IncR	Tn4401a
ST1284 (1)	*E. kobei*	*bla_OXA-48_*	*bla_ACT-9_*	*fosA-1*	IncL/M	Tn1999

^*a*^ Inc ND, incompatibility group not determined.

Based on the type of carbapenemase gene, the clone distribution was as follows. Among *bla*_VIM_-carrying ECC, 72.2% (13/18) isolates belonged to the most frequent clones (ST93, ST78, and ST50). The two *bla*_KPC_-positive strains were assigned to ST54 and ST93, respectively. The single isolate harboring the *bla*_OXA-48_ gene was identified as ST1284.

In addition to carbapenemase genes, all clones exhibited an extensive set of antimicrobial resistance genes, except for ST758 and ST1284, which had relatively fewer genes (Table 1). ESBL genes were presented in all clones, except in ST50, ST758, and ST1284. Interestingly, ST54 and ST145 (DLV ST78) displayed the presence of the *mcr-9* gene, associated with colistin resistance.

### Plasmid analysis

**Fig 3:**
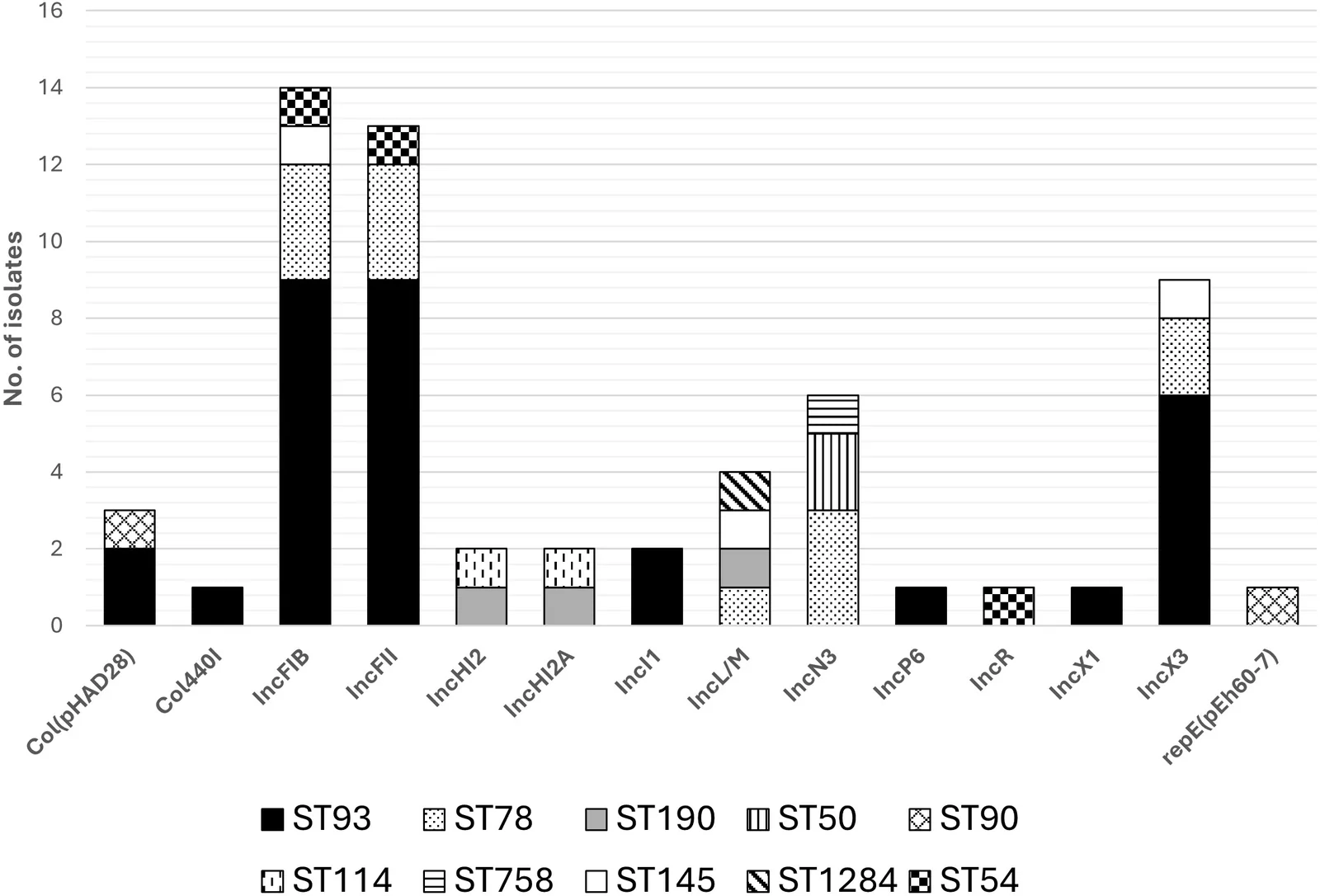
Incompatibility groups and Col plasmids of the carbapenem-resistant *Enterobacter cloacae* complex according to the sequence type.

Among the 21 ECC isolates, IncFIB, IncFII, and IncX3 were the most prevalent plasmids, accounting for up to 66.7% (*n* = 14), 62% (*n* = 13), and 42.8% (*n* = 9), respectively. At least one plasmid was found in all strains. Incompatibility groups and Col plasmids identified according to the ST in the 21 ECC isolates are shown in Fig. 3. Considering the type of clone, ST93 exhibited the highest diversity in plasmid types. In contrast, the ST50, ST758, and ST1284 isolates each possessed a single plasmid: IncN3 was detected in ST50 and ST758, whereas ST1284 harbored IncL/M.

The localization of carbapenem resistance genes on plasmids was also analyzed (Table 1). Carbapenem resistance determinants were found on four plasmid incompatibility (Inc) replicon groups: IncL/M, IncN, IncP, and IncR. Even though most *bla*_VIM_-carrying plasmids could not be assigned to known incompatibility groups (13/18 isolates), this gene was localized on IncL/M (three isolates) and IncN (two isolates) plasmids.

The strains harboring an IncL/M-type plasmid belonged to ST78, ST145, and ST190, respectively. IncN plasmids carrying *bla*_VIM-1_ were identified in the two ST50 isolates.

Regarding other carbapenem resistance genes, the *bla*_OXA-48_ gene (identified in ST1284 clone) was associated with the IncL/M. Plasmids carrying the *bla*_KPC_ gene belonged to two incompatibility groups: IncP6 (one isolate) and IncR (one isolate).

### Genetic environment of carbapenem resistance gene

**Fig 4:**
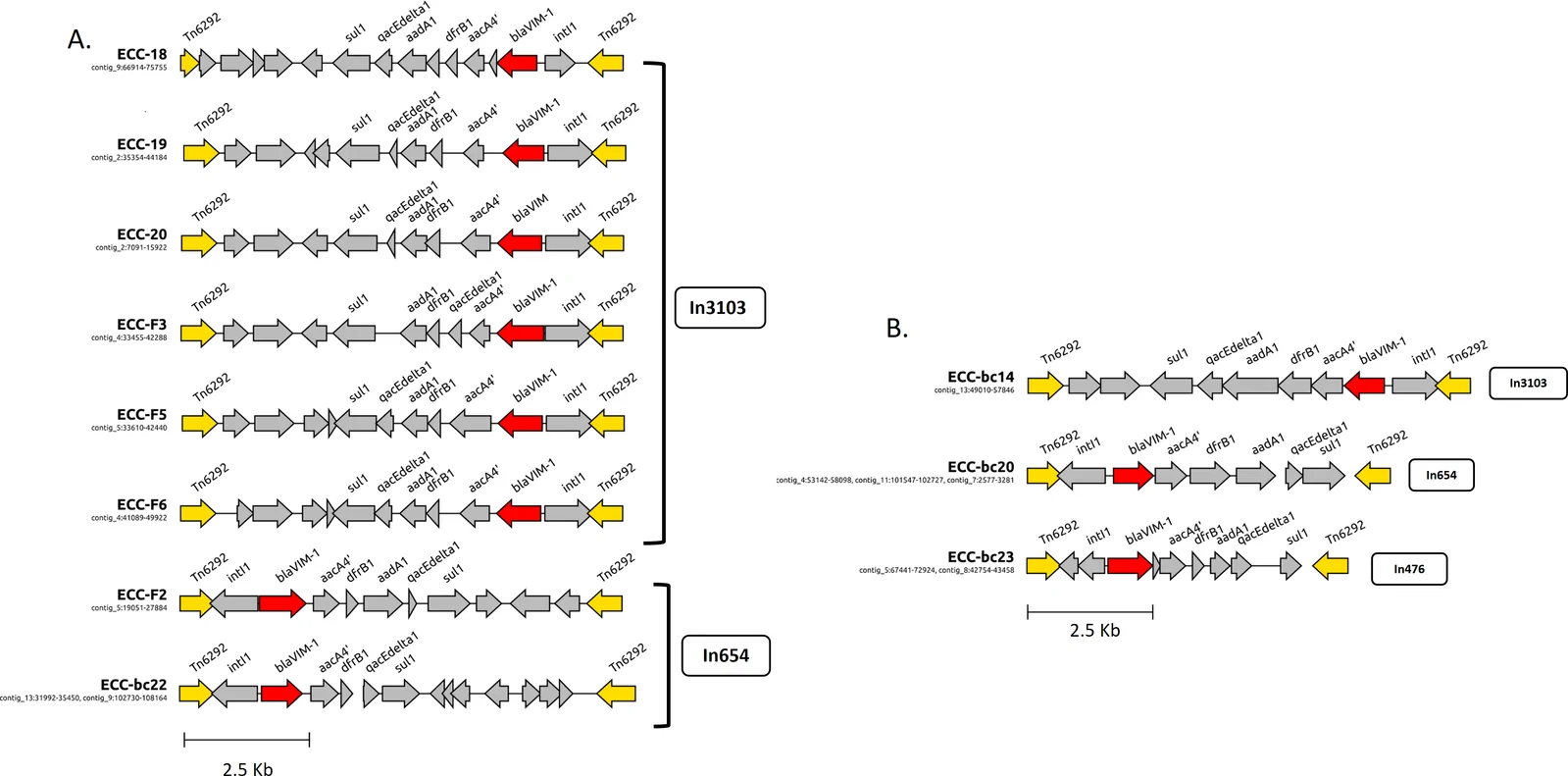
Schematic representation of mobile genetic elements responsible for the dissemination of *bla*_VIM-1_ genes in the predominant clones: (**A**) ST93 and (**B**) ST78 *Enterobacter cloacae* complex isolates, respectively.

Mobile genetic elements involved in the dissemination of carbapenem resistance determinants were also studied (Table 1). The *bla*_VIM-1_ gene was part of class 1 integrons in 17/18 isolates, including the integrons In3103 (*n* = 7), In654 (*n* = 4), In114 (*n* = 4), In476 (*n* = 1), and In624 (*n* = 1). Moreover, except in one strain, this class 1 integron was located within the Tn3-family transposon Tn6292. Schematic representations of mobile genetic elements responsible for the dissemination of *bla*_VIM-1_ genes in the predominant clones (ST93 and ST78) are presented in Fig. 4.

Notably, two copies of the *bla*_VIM_ gene were observed in one strain belonging to ST93 and another belonging to ST190. The ST93 isolate carried both *bla*_VIM_ genes within the same contig, and each gene was located within an In3103 integron embedded in the Tn6292 transposon. In the case of the strain ST190, the *bla*_VIM_ genes were found on separate plasmids: one IncL/M-type plasmid and the other non-typeable. Both genes were also part of a transposon Tn6292, containing a class 1 integron (In624 and In708, respectively).

The *bla*_KPC-3_ gene was located on a Tn3-family transposon Tn4401a, while *bla*_KPC-2_ was flanked by ISKpn6 upstream and ISKpn27 downstream, in association with multiple transposase sequences and a resolvase gene (*tnpR*). The contig harboring *bla*_OXA-48_ was highly similar (>99% identity) to IncL/M plasmid carrying *bla*_OXA-48_ within a Tn1999 transposon (pOXA48a, accession number JN626286.1).

### Phylogenetic relationship

**Fig 5:**
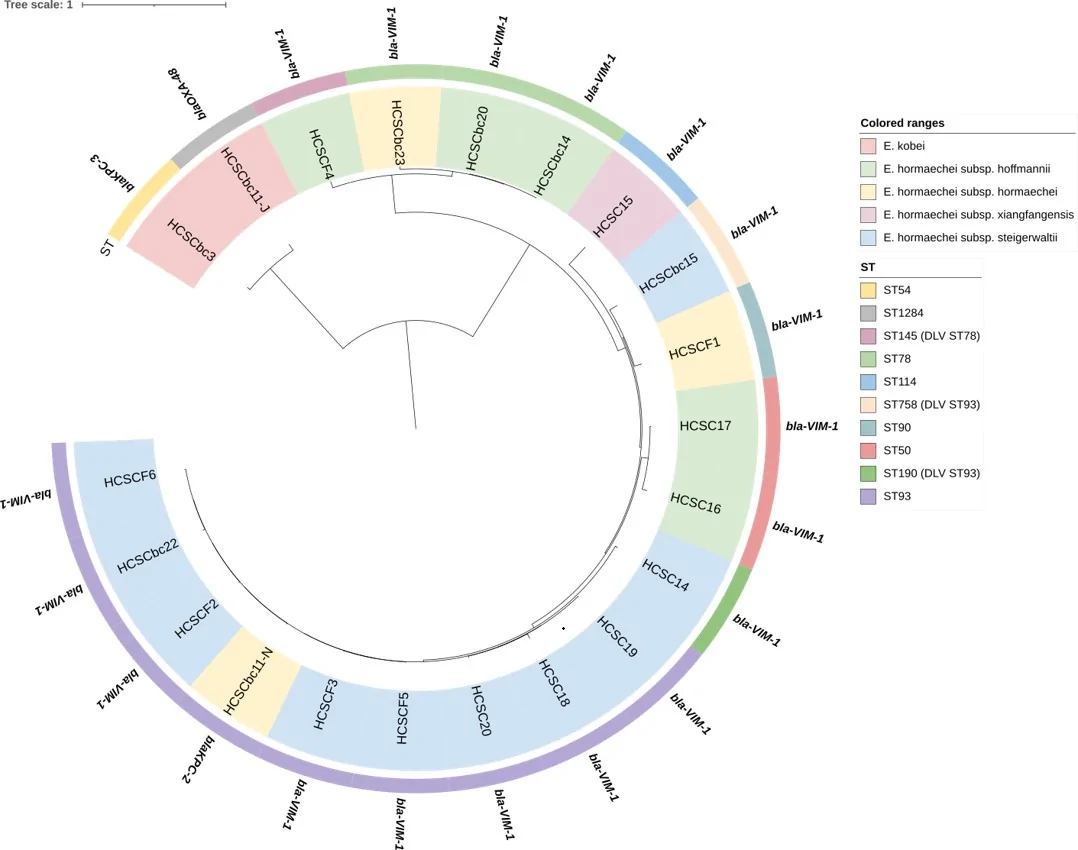
Single-nucleotide polymorphism (SNP) tree of the 21 carbapenemase-resistant *Enterobacter cloacae* complex isolates. From inside to outside, each ring sequentially displays *Enterobacter cloacae* complex (ECC) species, the sequence types (STs), and carbapenemase-encoding genes identified.

**Fig 6:**
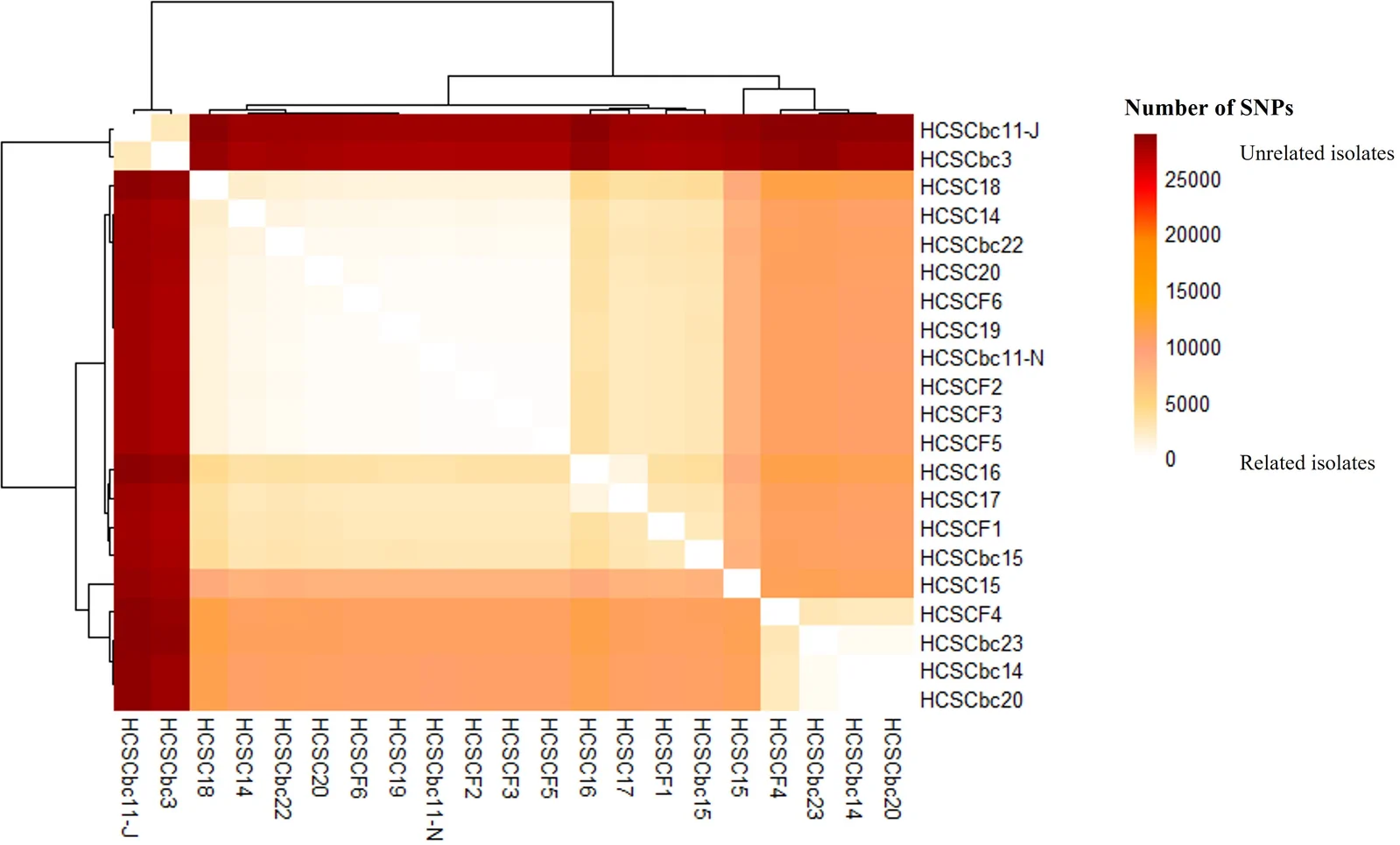
Phylogenetic tree and SNP matrix of 21 carbapenemase-resistant *Enterobacter cloacae* complex isolates. Gradient of SNP differences between isolates is detailed.

SNP-based phylogenetic analysis demonstrated that isolates belonging to the same ST clustered together, with fewer than 1,500 single-nucleotide polymorphism (SNP) differences (Fig. 5 and 6).

All isolates from the same group were closely related to each other, while a clear genetic divergence was observed between strains from distinct clusters.

All 10 *E. hormaechei* subsp. *steigerwaltii* isolates were assigned to the most common ST in our study, ST93 (DLV ST93). Conversely, the remaining species did not contain a predominant ST but instead displayed a diversity of STs. Among the five *E. hormaechei* subsp. *hoffmannii* isolates, two frequent clones were identified: ST78 (*n* = 3) and ST50 (*n* = 2). *E. hormaechei* subsp. *kobei* was represented by ST1284 (*n* = 1) and ST54 (*n* = 1). *E. hormaechei* subsp. *hormaechei* comprised three different STs (ST93, ST70, and ST90). Lastly, the *E. hormaechei* subsp. *xiangfangensis* isolate belonged to ST114.

## DISCUSSION

Colonization by specific bacterial species, particularly multidrug-resistant strains, has been consistently associated with an increased risk of subsequent infection by the same organisms (22, 23). In this context, colonization with ECC may act as a reservoir for both infection and transmission (9, 24). This study about carbapenemase-producing ECC strains colonizing hospitalized patients highlights the potential for colonized individuals to contribute to the spread of these bacteria within healthcare settings. The persistence and dissemination of ECC in healthcare environments are strongly influenced by its high genetic diversity and remarkable capacity to acquire antimicrobial resistance genes (3, 8). Effective infection control and surveillance are essential to prevent outbreaks and protect vulnerable patients.

We performed WGS to genetically characterize 21 carbapenem-resistant ECC strains isolated from colonized patients at Hospital Clínico San Carlos (HCSC). The most common carbapenemase gene from our collection was *bla*_VIM_ (85.7%), while *bla*_KPC_ and *bla*_OXA-48_ were only identified in two and three strains, respectively. Similar results were obtained in a recent study in Spain including 334 carbapenem-resistant ECC strains (25), with *bla*_VIM_ being the most prevalent gene. Furthermore, Matsumura et al. (26) reported notable geographic patterns in the distribution of carbapenemase genes among clinical carbapenemase-producing *Enterobacter* spp. isolates, with *bla*_VIM_ being mainly found in Europe.

*E. hormaechei* subsp. *steigerwaltii* (ST93), followed by *E. hormaechei* subsp. *hoffmannii* (ST78 and ST50), were the most prevalent species and STs circulating among the *bla*_VIM_-harboring strains. These clones are considered high-risk clones within the ECC, as they have been previously reported worldwide and are associated with different carbapenemases (5, 8, 12, 14, 26–28). In our collection, we also identified a single isolate of *E. hormaechei* subsp. *hormaechei* ST93 harboring *bla*_KPC-2_. However, the *E. kobei* strain carrying *bla*_KPC-3_ belonged to the ST54 clone. To our knowledge, the *bla*_KPC-3_ gene can be found in ECC, but it is not commonly associated with the ST54 sequence type. Other sequence types, such as ST171, are more frequently linked to *bla*_KPC-3_ globally (26, 27). Finally, the isolate harboring *bla*_OXA-48_ in this study belonged to *E. kobei* ST1284. This finding represents a novel observation not previously described in the reviewed literature. Typically, this gene is associated with international high-risk clones such as ST66, ST171, and ST78 (8).

Interestingly, two minority STs exhibited the presence of the *mcr-9* gene. *E. kobei* ST54 co**-**harbored *bla*_KPC-3_ and *mcr-9*, and *E. hormaechei* subsp. *hoffmannii* ST145 (DLV ST78) carried *bla*_VIM-1_ and *mcr-9*. The coexistence of mobile colistin resistance *mcr-9* with metallo-β-lactamase genes in carbapenem-resistant ECC is a significant concern, as it signals resistance to two last-resort antibiotics (colistin and carbapenems), highlighting the risk of difficult-to-control hospital outbreaks (29). A national surveillance study in the Czech Republic identified three *E. kobei* ST54 isolates carrying the *mcr* gene. These strains exhibited multidrug resistance, but the study did not confirm the presence of *bla*_KPC_ in these specific ST54 isolates (30). Other studies have reported *E. hormaechei* strains (ST93) co-harboring *mcr* and *bla*_KPC-2_, often on separate plasmids (31). Our ST54 isolate had the *mcr-9* and *bla*_KPC-3_ genes on IncF and IncR plasmids, respectively. Nevertheless, the *mcr-9* gene can also be integrated into the chromosome of ECC isolates (30). Accurately, we found that the ST145 (DLV ST78) isolate carried the *mcr-9* gene on a Tn602 integrated into the chromosome and an IncL/M plasmid containing *bla*_VIM-1_. The coexistence of *bla*_VIM-1_ and *mcr-9* has also been described in a multidrug-resistant ECC ST78 strain in the Netherlands (32).

Antimicrobial resistance is disseminated not only through clonal expansion of bacterial strains but also via horizontal gene transfer mediated by mobile genetic elements. A review published by Kopotska et al. (33) describes a wide distribution of plasmid incompatibility (Inc) replicon groups linked to carbapenem resistance genes. Plasmid typing in our investigation revealed four plasmid types—IncL/M (with *bla*_VIM-1_ or *bla*_OXA-48_), IncN (*bla*_VIM*-*1_), IncP (*bla*_KPC-2_), and IncR (*bla*_KPC-3_)—associated with these resistance determinants. These results are consistent with previous studies. Matsumura et al. found *bla*_VIM_ genes on IncL/M and IncN plasmids in clinical *Enterobacteriaceae* (34). Conversely, plasmid replicon types, such as IncP and IncR, are occasionally reported in KPC-producing *K. pneumoniae* (33).

Notably, IncP plasmids harboring *bla*_KPC-2_ have also been found in *Enterobacteriaceae* isolates recovered from hospital sewage (35). Additionally, *bla*_OXA-48_-carrying IncL/M plasmid has been extensively described across various bacterial species and geographic regions (8, 33).

It is widely known that *bla*_VIM_ genes are frequently identified in class 1 integrons (14, 26, 34, 36). Even though our study demonstrated a diversity of integrons involved in the dissemination of *bla*_VIM_ genes—In3103, In1114, and In654 being the most frequent—all of them were classified as class 1 integrons. In3103 harboring *bla*_VIM_ has previously been reported in ECC (14, 26, 34). However, in Spain, it is more common to find ST78 strains harboring In624 (26, 37), suggesting a regional prevalence of this integron in carbapenem-resistant isolates. The present study identified In624 in two DLV strains belonging to the most prevalent STs, ST93, and ST78. On the other hand, the second most common integrons in our study (In1114 and In654) are rarely detected in ECC as a carrier of *bla*_VIM_ (14, 26, 34, 37).

Noteworthily, in every isolate analyzed, the *bla*_VIM_ gene was not only located on a class 1 integron but also integrated into a Tn3-family transposon, Tn6292, underscoring its high potential for horizontal gene transfer and widespread dissemination. To our knowledge, this transposon has not been described as a carrier of *bla*_VIM_; however, *bla*_KPC_ and *bla*_NDM_ genes have been linked to this mobile element, Tn6292, in clinical isolates of *Enterobacteriaceae* (38, 39). In fact, we have continued to detect ECC strains harboring the *bla*_NDM_ gene in our hospital, and sequence analysis reveals that this gene is located within the Tn6292 transposon. Additionally, the Tn6292 transposon also commonly contained the *qnrS* gene (38). In our study, the two ST50 isolates co-harbored *bla*_VIM_ and *qnrS* within the Tn6292 transposon.

The genetic context of the remaining carbapenemase genes differed from that of *bla*_VIM_. The *bla*_KPC-3_ gene was associated with the Tn4401, whereas *bla*_OXA-48_ was consistently linked to Tn1999. Our findings are in accordance with previous studies on the genetic environments of the carbapenemase genes (26, 40).

The *bla*_OXA-48_ gene is mainly disseminated via the Tn1999 transposon (41), typically embedded in highly transferable and stable IncL/M plasmids such as pOXA48. In contrast, *bla*_KPC_ is primarily mobilized by Tn4401, a transposon responsible for carbapenem resistance in *Klebsiella pneumoniae* and other *Enterobacteriaceae* (40). In our study, the contig containing *bla*_KPC-2_ was incomplete but showed 99.88% homology with the IncP6 plasmid. Additionally, *bla*_KPC-2_ was flanked upstream by ISKpn6 and downstream by ISKpn27, along with several transposase elements and a resolvase gene. These mobile genetic elements associated with *bla*_KPC-2_ have been previously described in several bacterial species (33). Although ISKpn27 is not part of the canonical Tn4401 structure (which typically includes transposase and resolvase genes and two insertion sequences, IS*Kpn6* and IS*Kpn7*, in addition to *bla*_KPC-2_), it may contribute to *bla*_KPC_ mobilization, as highlighted by Feng et al. (42). Furthermore, Tn4401 could incorporate additional insertion sequences during its evolution, reinforcing the potential role of ISKpn27 in such structural variants (40).

In conclusion, analysis of the genetic context of carbapenemase genes underscores the utility of WGS in ECC epidemiology, demonstrating that the *bla*_VIM-1_ gene is associated with a class 1 integron located in Tn6292. This study provides insight into both the clonal responsible for *bla*_VIM-1_ dissemination and the mobile genetic elements underlying this process.

## Data Availability

The genome of the isolates is available at NCBI (PRJNA1366112).

## SUPPLEMENTAL MATERIAL

Supplemental material spectrum.04162-25-s0001.docx Table S1.

ASM does not own the copyrights to Supplemental Material that may be linked to, or accessed through, an article. The authors have granted ASM a non-exclusive, world-wide license to publish the Supplemental Material files. Please contact the corresponding author directly for reuse.
