# Supplementary material for: Mobilization of blaVIM genes via the Tn6292 transposon among carbapenem-resistant Enterobacter cloacae complex isolates from colonized patients in a Spanish hospital
Source: Microbiol Spectr. 2026 Jul 21;14(9):e04162-25. doi: 10.1128/spectrum.04162-25 (PMC13532250; doi:10.1128/spectrum.04162-25)
Supplement: Supplementary file 1 [file spectrum.04162-25-s0001.docx]

**SUPPLEMENTAL MATERIAL**

**Table S1.** Sequencing Metrics: Genome Coverage and N50 Values.

| **Strain** | **Coverage** | **N50** |
| --- | --- | --- |
| ECC-14 | 85x | 4.81Mb |
| ECC-15 | 79x | 4.79Mb |
| ECC-16 | 13x | 4.84Mb |
| ECC-17 | 39x | 4.84Mb |
| ECC-18 | 12x | 4.78Mb |
| ECC-19 | 62x | 4.79Mb |
| ECC-20 | 29x | 4.79Mb |
| ECC-bc11-J | 59x | 4.78Mb |
| ECC-bc11-N | 7x | 1.31Mb |
| ECC-bc14 | 20x | 1.36Mb |
| ECC-bc15 | 122x | 4.66Mb |
| ECC-bc20 | 10x | 536Kb |
| ECC-bc22 | 36x | 4.78Mb |
| ECC-bc23 | 81x | 4.63Mb |
| ECC-bc3 | 26x | 823Kb |
| ECC-F1 | 201x | 4.8Mb |
| ECC-F2 | 38x | 4.79Mb |
| ECC-F3 | 128x | 4.79Mb |
| ECC-F4 | 40x | 4.80Mb |
| ECC-F5 | 232x | 4.79Mb |
| ECC-F6 | 27x | 4.79Mb |
